# Gestational Mercury Vapor Exposure and Diet Contribute to Mercury Accumulation in Neonatal Rats

**DOI:** 10.1289/ehp.8754

**Published:** 2006-01-13

**Authors:** Daniel L. Morgan, Herman C. Price, Reshan Fernando, Sushmita M. Chanda, Robert W. O’Connor, Stanley S. Barone, David W. Herr, Robert P. Beliles

**Affiliations:** 1 National Institute of Environmental Health Sciences, National Institutes of Health, Department of Health and Human Services, Research Triangle Park, North Carolina, USA; 2 ManTech Environmental Technology Inc., Research Triangle Park, North Carolina, USA; 3 Research Triangle Institute, Research Triangle Park, North Carolina, USA; 4 National Health and Environmental Effects Research Laboratory, U.S. Environmental Protection Agency, Research Triangle Park, North Carolina, USA; 5 U.S. Environmental Protection Agency, Washington, DC, USA

**Keywords:** brain, diet, gestational exposure, kidney, liver, Long-Evans rats, mercury, neonate

## Abstract

Exposure of pregnant Long-Evans rats to elemental mercury (Hg^0^) vapor resulted in a significant accumulation of Hg in tissues of neonates. Because elevated Hg in neonatal tissues may adversely affect growth and development, we were interested in how rapidly Hg was eliminated from neonatal tissues. Pregnant rats were exposed to 1, 2, or 4 mg Hg^0^ vapor/m^3^ or air (controls) for 2 hr/day from gestation day 6 (GD6) through GD15. Neonatal brain, liver, and kidney were analyzed for total Hg at various times between birth and postnatal day 90 (PND90). Milk was analyzed for Hg between birth and weaning (PND21). Before weaning, the Hg levels in neonatal tissues were proportional to maternal exposure concentrations and were highest in kidney followed by liver and then brain. There was no elimination of Hg between birth and weaning, indicating that neonates were exposed continuously to elevated levels of Hg during postpartum growth and development. Consumption of milk from exposed dams resulted in a slight increase in kidney Hg concentration during this period. Unexpectedly, neonatal Hg accumulation increased rapidly after weaning. Increased Hg was measured in both control and exposed neonates and was attributed to consumption of NIH-07 diet containing trace levels of Hg. By PND90, tissue Hg levels equilibrated at concentrations similar to those in unexposed adult Long-Evans rats fed the same diet. These data indicate that dietary exposure to trace amounts of Hg can result in a significantly greater accumulation of Hg in neonates than gestational exposure to high concentrations of Hg^0^ vapor.

Elemental (metallic) mercury (Hg^0^) is a ubiquitous environmental contaminant that can cause serious adverse health effects [Agency for Toxic Susbtances and Disease Registry (ATSDR) 1997]. Sources of Hg^0^ exposure include dental amalgams, fossil fuel emissions, chloralkalai production, thermometers, incandescent lights, medical waste incineration, cremation, and Hg used in ritualistic practices (Counter and Buchanan 2004). Because Hg^0^ is highly volatile, the primary route of human exposure is by inhalation of the vapor. Inhaled Hg^0^ vapor is readily absorbed from the respiratory tract into the systemic circulation (Hursh et al. 1976).

In the pregnant female, Hg^0^ has been shown to penetrate the placental barrier (Clarkson et al. 1972; Khayat and Dencker 1982; Lutz et al. 1996; Warfvinge et al. 1994; Yoshida et al. 1990) and to be taken up by fetal tissues. As in maternal tissues, oxidation of Hg^0^ in the fetal tissues converts it to mercuric mercury (Hg^2+^), which is much less likely to recross the placental barrier. Thus, oxidation may result in an accumulation of Hg in the fetal tissues (Baranski and Szymczyk 1973; Campbell et al. 1992) and has raised concerns regarding the potential toxicity for the fetus and the neonate.

Data on the elimination of inorganic Hg in the neonate after gestational exposure are sparse, yet this information could be important in developing strategies to prevent or limit postnatal Hg toxicity. In the adult animal, the elimination pathway for inorganic Hg is complex, with a half-life that varies depending upon the tissue type and the time after exposure [see International Programme on Chemical Safety (IPCS) 1991]. Evaluation of Hg elimination in neonatal animals exposed *in utero* is complicated further because the animals are growing rapidly, resulting in dilution of the Hg retained in tissues. In addition, the neonate may receive additional exposure to Hg from maternal milk before weaning and from food and water after weaning. We investigated the retention and elimination of Hg from neonatal tissues because of the potential adverse effects of elevated Hg on neonatal growth and development. Neonatal liver, kidney, and brain were examined because these are major deposition sites for Hg and are the most common sites of Hg toxicity (IPCS 1991).

## Materials and Methods

### Animals.

Timed-pregnant Long-Evans rats (225–250 g; Charles River Breeding Laboratories, Portage, MI) were received on gestation day 2 (GD2), where GD0 is defined as the day mating was confirmed (presence of vaginal plug). Rats were individually housed, and food (NIH-07; Zeigler Brothers, Inc., Gardners, PA) and water (deionized, filtered tap water) were provided *ad libitum* except during exposure. On GD4, rats were weighed and randomized into control and treatment groups. Body weights of rats were recorded daily beginning on GD5 through postnatal day 1 (PND1). PND0 was defined as the day of birth (between 0700 hr and 1600 hr), and PND1 was defined as the day after birth.

Neonates were housed with dams until weaned at PND21. On PND21, neonates were separated from the dams and group housed with littermates (three per cage). Weaned neonates received NIH-07 feed and filtered tap water *ad libitum*. Neonates from air-exposed control dams were isolated from neonates from Hg^0^-exposed dams throughout the study. Cages were thoroughly washed with hot soapy water in a cage washer to remove traces of Hg.

The NIH-07 rodent feed used in this study was certified by the manufacturer (Zeigler Brothers, Inc.) to contain < 0.025 ppm (0.025 μg/g) Hg. Independent analyses of the NIH-07 feed used in this study agreed with the certification. A method for differentiating organic and inorganic Hg was not available. Water samples were collected from the automatic watering system that supplied the rat cages. The water lines were flushed and filled with fresh water, and 10-mL samples were collected 24 hr later. The total Hg content in all water samples was less than the 6.250 pg/mL limit of quantification.

This study was conducted under federal guidelines for the use and care of laboratory animals (National Academy of Sciences 1996) and was approved by the National Institute of Environmental Health Sciences and U.S. Environmental Protection Agency Animal Care and Use Committees. Animals were housed in a humidity- and temperature-controlled, HEPA-filtered, mass-air-displacement room in facilities accredited by the American Association for Accreditation of Laboratory Animal Care. Animal rooms were maintained with a light/dark cycle of 12 hr (light from 0700 to 1900 hr). Sentinel animals housed in the animal facility as part of an ongoing surveillance program for parasitic, bacterial, and viral infections were pathogen-free throughout the study. The animals were treated humanely and with regard for alleviation of suffering during these studies.

### Mercury vapor (Hg^0^) exposure system.

#### Generation and monitoring.

Inhalation exposures were conducted at the National Institute of Environmental Health Sciences inhalation facility. Hg^0^ vapor was generated by passing conditioned air (HEPA filtered, charcoal scrubbed, temperature and humidity controlled) through a flask containing 10–20 g of liquid Hg^0^. The flask containing Hg^0^ was immersed in a temperature-controlled water bath maintained at approximately 2°C above ambient. The resulting Hg^0^ vapor was diluted and delivered to the exposure system at a controlled rate using mass flow controllers.

Control animals were exposed to conditioned air in a stainless-steel, 52-port nose-only exposure system (Lab Products, Rockville, MD). An analogous nose-only exposure system, constructed of polycarbonate, was used for Hg^0^ vapor exposures to 1, 2, or 4 mg/m^3^. The airflow through both systems was maintained at approximately 12 L/min. Each experiment consisted of a group of animals exposed to one Hg^0^ vapor concentration and a concurrent air-exposed control group. Exposure concentrations were measured from the nose-only system once every 15–30 min, and air samples from the room, scrubber, and the exposure system enclosure were analyzed once every hour. Air samples were analyzed using a Jerome model 431-X mercury analyzer (Arizona Instruments Phoenix, AZ) that is specific for Hg^0^ vapor.

#### Animal exposures.

Pregnant rats were exposed by nose-only inhalation to avoid contamination of the fur and subsequent oral and dermal exposure to Hg^0^ vapor. Rats were exposed to either Hg^0^ (1, 2, or 4 mg/m^3^) or conditioned air (controls) for 10 consecutive days on GD6 through GD15 as described previously (Morgan et al. 2002).

### Tissue collection for Hg analyses.

Neonates from each of five litters (one pup per litter) per exposure group were randomly selected and euthanized on PND1, PND14, PND21, PND45, and PND90. Neonates were not separated by sex. We selected brain, liver, and kidney for Hg analyses because in a previous study these tissues accumulated the highest levels of Hg in exposed pregnant rats (Morgan et al. 2002). Neonatal brain, liver, and both kidneys were collected at each time point and stored at −20°C until analyzed for total Hg. Before collection of each tissue sample, stainless-steel dissection instruments were rinsed in 10% hydrochloric acid and deionized water to reduce cross-contamination with Hg. Tissues were placed in acid-washed glass vials prepared in a class 100 clean room and stored at −20°C until processed for Hg analysis.

### Milk collection.

Milk from Hg^0^ exposed dams can contribute to the neonatal body burden before weaning (PND21). Milk was collected from Hg^0^-treated dams on PND4, PND10, PND14, and PND21 and from control dams on PND14 by a modification of the method of Oskarsson et al. (1995). Briefly, pups were separated from dams for approximately 4 hr before milk collection to allow milk to accumulate. Dams were sedated with keta-mine/xylazine [intraperitoneal (ip) 100 mg/kg] and were given oxytocin (ip, 1 IU; 0.2 mL of a 5 IU solution) about 5 min before collection. Milk was expressed manually into tared, acid-washed vials. Samples were weighed and stored at −20°C until processed for Hg analysis.

### Hg analyses.

Mercury analyses were conducted by the Research Triangle Institute. Tissue samples were weighed and homogenized in deionized water. Homogenates were digested with sulfuric acid/nitric acid (30/70) overnight at 70°C in sealed vials. The next morning, samples were cooled, diluted with concentrated hydrochloric acid (trace metal grade), and neutralized. Digested samples were diluted to volume with deionized water and analyzed for total Hg by cold-vapor atomic fluorescence spectrometry as described previously (Levine et al. 2002). Milk samples were prepared the same as tissue samples without the homogenization step. Sample preparation methods were developed and validated for each tissue type. The percent recovery from Hg-spiked samples was determined for each tissue. Method detection limits (MDLs) for neonatal tissues were 0.432 ng/g brain, 0.851 ng/g liver, 6.6 ng/g kidney, and 1.99 pg/mL milk. All Hg species present in tissues were converted to divalent Hg^2+^ during sample processing; thus, Hg content represents total Hg species. Methods were not available for measuring individual Hg species in tissues.

Because neonatal tissues were rapidly growing during the evaluation period (PND1–PND90), it was necessary to account for the increasing organ mass with age when calculating Hg content. At each time point, organ weights were measured and the Hg content was calculated as concentration (nanograms Hg per gram of tissue) and as Hg per whole organ (nanograms Hg per gram of tissue × total organ weight).

### Statistical analyses.

We used analysis of variance procedures and Dunnett’s multiple comparison test (Sokal and Rohlf 1969) to assess the significance of differences (*p* < 0.05) among organ weights and tissue Hg levels.

## Results

### Mercury analyses.

#### Milk.

Concentrations of Hg in milk from lactating dams were related to exposure concentration. Hg concentrations decreased with time after exposure, with the highest concentrations measured on PND4 and the lowest concentrations detected on PND14 and PND21 (Table 1). The highest Hg concentrations were found in milk from dams exposed to 4 mg/m^3^ Hg^0^ vapor, and milk from dams exposed to 2 and 1 mg/m^3^ had proportionately lower amounts of Hg. Hg concentrations in milk from air-exposed dams were below the limit of quantification (0.663 ng/g milk).

#### Brain.

**Figure 1:**
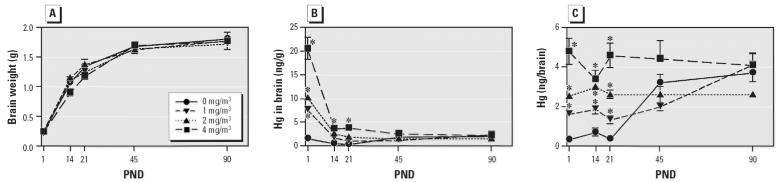
Effect of *in utero* Hg vapor exposure (0, 1, 2, or 4 mg/m^3^) on neonatal brain weight (*A*), Hg concentration in brain (ng/g; *B*), and total Hg per brain (ng/brain; *C*) from PND1 to PND90. Data represent mean ± SE. *Significantly different from controls (*p* < 0.05).

The most rapid brain growth occurred before PND21 (Figure 1A). Brain weights in all treatment groups increased approximately 5- to 6-fold from PND1 to PND 21 and another 5- to 6-fold from PND21 to PND90. We found no statistically significant differences in brain weights between treatment groups and controls at any time points.

The Hg concentrations (nanograms per gram of tissue) in neonatal brain were related to exposure concentration and were highest on PND1 (Figure 1B). The Hg concentrations in the brains of treated groups decreased most rapidly between birth and weaning, corresponding to the time of greatest brain growth. Between weaning at PND21 and PND90, brain Hg concentrations in the treated groups decreased only slightly. Hg concentrations in control brain began to increase after weaning, and by PND45 and PND90 Hg concentrations in control brain had attained the same level as in treated brain.

Brain Hg content was also expressed as nanograms of Hg per whole brain to adjust for organ growth. Whole-brain Hg content was exposure-concentration related and remained relatively unchanged in all treated groups before PND21 (Figure 1C). Brain Hg content in the 2 and 4 mg/m^3^ dose groups remained relatively unchanged from PND 1 to PND90. However, brain Hg in the control and 1 mg/m^3^ dose groups began to increase after PND21, and by PND90 Hg levels were not significantly different between the controls and treated groups. The MDL for Hg in neonatal rat brain was 0.432 ng/g tissue.

#### Liver.

**Figure 2:**
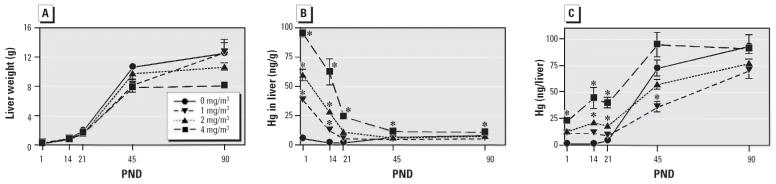
Effect of *in utero* Hg vapor exposure (0, 1, 2, or 4 mg/m^3^) on neonatal liver weight (*A*), Hg concentration in liver (ng/g; *B*), and total Hg per liver (ng/liver; *C*) from PND1 to PND90. Data represent mean ± SE. *Significantly different from controls (*p* < 0.05).

Liver weights in all exposed groups increased approximately 7-fold from PND1 to PND21 and 6.5-fold between PND21 and PND90 (Figure 2A). The most rapid increase in liver weights occurred after PND21 for all groups. We found no significant differences in liver weights between treated and control groups at all time points. Although liver weights in the 4 mg/m^3^ dose group appeared less than that of controls at PND90, this difference was not statistically significant.

Before weaning, liver Hg concentrations (nanograms Hg per gram of tissue) were directly related to maternal exposure concentrations and were all significantly different at PND1, PND14, and PND21 (Figure 2B). Liver Hg concentrations were highest at PND1 and decreased rapidly until PND21. Concentrations changed only slightly after PND21, and at PND45 and PND90 the Hg concentrations in the 1 and 2 mg/m^3^ exposure groups were not significantly different from controls. Liver Hg concentration also decreased with age in the 4 mg/m^3^ exposure group but remained significantly greater (*p* < 0.05) than controls at PND45 and PND90.

When calculated as Hg per whole organ (nanograms per liver), Hg levels in all groups remained relatively constant from PND1 to PND21. After PND21, there was an abrupt increase in Hg levels of all groups, especially controls (Figure 2C). Hg levels in the 1, 2, and 4 mg/m^3^ exposure groups remained related to exposure concentration through PND45, but by PND90 all treatment groups had equilibrated at about the same Hg level. After weaning, the greatest increases in Hg occurred in the controls, and by PND90 there were no significant differences in liver Hg content between controls and exposure groups. The MDL for neonatal rat liver was 0.851 ng/g tissue.

#### Kidney.

**Figure 3:**
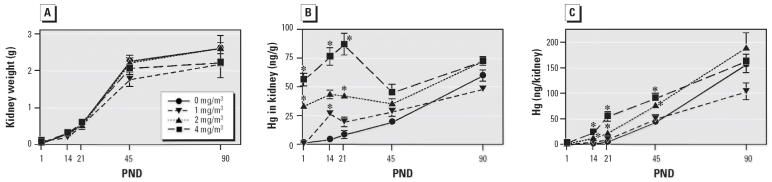
Effect of *in utero* Hg vapor exposure (0, 1, 2, or 4 mg/m^3^) on neonatal kidney weight (*A*), Hg concentration in kidney (ng/g; *B*), and total Hg per kidney (ng/kidney; *C*) from PND1 to PND90. Data represent mean ± SE. *Significantly different from controls (*p* < 0.05).

Kidney weights (left and right combined) in all groups increased with neonatal age with the largest increase (about 10-fold) occurring before PND21 (Figure 3A). Kidney weights increased 4- to 5-fold between PND21 and PND90. No statistically significant differences in kidney weights were found between treatment groups at any time point.

In general, between birth and PND21, Hg concentrations (nanograms per gram of tissue) in neonatal kidneys were exposure concentration-related and were significantly greater than concentrations in control kidneys (Figure 3B). In contrast to brain and liver, kidney Hg concentrations increased in all dose groups between birth and PND21. Hg concentrations in the control, 1 mg/m^3^, and 2 mg/m^3^ groups continuously increased between PND1 and PND90. Kidney Hg concentrations in the 4 mg/m^3^ group decreased after PND 21 and were not significantly different from controls and other treatment groups at PND45 and PND90.

When expressed as Hg per whole kidney, the Hg content increased continuously with age in all groups with the highest levels attained at PND90 (Figure 3C). In general, Hg levels were exposure-concentration related at PND14, PND21, and PND45; however, by PND90 there were no significant differences between controls and treated groups. The MDL for kidney was 6.6 ng/g tissue.

### Food and water consumption.

Because we did not measure food and water consumption during the study, the amount of Hg contributed by the diet was estimated. Reported food and water consumption data for bodyweight–matched and age-matched Long-Evans rats were obtained from Charles-River Laboratories (Wilmington, MA). Because the Hg levels in the food and water were detectable but below the MDL, they were assigned a value midway between the MDL and zero (1/2 MDL). The Hg content was estimated to be 0.0125 μg/g feed and 3.12 pg/mL water. These data were used to estimate the amount of Hg consumed in the food and water per day. For the purposes of this estimation, absorption of ingested Hg was assumed to be 100% with no elimination. Based on these calculations, we estimated that weanling rats could accumulate approximately 1.75–2 μg Hg by PND45 and approximately 8.5–10 μg Hg by PND90.

The amount of dietary Hg accumulated by neonates between weaning and PND90 was inversely proportional to the amount accumulated from gestational exposure (Table 2). Tissues from control neonates (no gestational Hg exposure) had the lowest Hg content at weaning and accumulated the greatest amount of Hg from diet. In control animals, the Hg content increased 10-fold in brain, 22-fold in liver, and 32-fold in kidney by PND90. In control and Hg-exposed neonates, accumulation of Hg was greatest in kidney, followed by liver, and was lowest in brain. By PND90, the Hg content of brain, liver, and kidney in control animals was similar to that in brain, liver, and kidney of exposed animals.

## Discussion

Exposure of pregnant rats to Hg^0^ vapor resulted in an accumulation of Hg in fetuses and retention of elevated Hg levels in neonates. Hg levels in neonatal tissues were proportional to maternal Hg^0^ vapor exposure concentrations. At birth, neonatal Hg concentrations were highest in kidney, intermediate in liver, and lowest in brain. Between birth and weaning, the neonates were growing rapidly, and the Hg concentrations decreased in brain and liver because of growth dilution. Brain mass increased much faster than that of liver, resulting in a more rapid dilution of the Hg concentration in the brain. Although the Hg concentration decreased, the total amount of Hg per organ did not change, indicating that there was no elimination of Hg from liver and brain. In kidney, both the Hg concentration and the total Hg per kidney increased, indicating an increased accumulation of Hg.

The lack of Hg elimination in brain and liver and the increased accumulation of Hg in kidney suggested that postnatal elimination was confounded by dietary Hg uptake. After birth, the neonates were no longer exposed to Hg in maternal blood; however, milk from the exposed dams provided a potential source for Hg uptake. Milk from dams contained exposure concentration-related amounts of Hg. Concentrations of Hg in milk decreased with time after parturition. Consumption of milk containing the highest concentration of Hg resulted in an increase in Hg in the kidney, whereas total Hg remained stable in liver and brain. The Hg species in milk were not determined but were assumed to be primarily inorganic Hg because there is little evidence of Hg methylation in tissues (IPCS 1991). Although bacteria in the mouth and gastrointestinal tract can metabolize inorganic Hg to methylmercury (Ludwicki 1989), this contribution to body burden would be low. A predominance of inorganic Hg would explain the low uptake in the presence of relatively high concentrations in milk, because inorganic Hg is not absorbed well from the gastrointestinal tract (IPCS 1991).

An unexpected finding in this study was the significant increase in Hg uptake that occurred when neonates were placed on the NIH-07 rodent diet. Because the Hg concentrations were below the MDLs in food and water, we did not expect dietary Hg to contribute significantly to the body burden relative to the amount contributed by gestational Hg^0^ exposure. However, we estimated that weanling rats could consume up to 10 μg Hg by PND90 based on average age-adjusted food consumption data and the assumption that Hg concentrations in feed were half the MDLs, or 0.0125 ppm. This dietary contribution could account for the increased tissue Hg levels. Water consumption may also have contributed a smaller amount to the Hg body burden. An estimate of the Hg contribution from water was not determined because accurate water consumption data were not available.

Certified laboratory animal diets were recently reported to contain levels of methyl-mercury high enough to affect study results, even though the levels were below the supplier’s detection limit of 0.02 ppm (Weiss et al. 2005). Methylmercury contamination of the diets was associated with fishmeal added as the protein source. Diets containing casein as the protein source did not contain Hg. The NIH-07 diet we used in the present study contained 10% fishmeal, and the Hg content was < 0.025 ppm. The Hg in the rodent diet was most likely methylmercury associated with fishmeal. About 95% of ingested methyl-mercury can be absorbed from the gastrointestinal tract (Goldman et al. 2001; Magus 1987) and could accumulate in neonatal tissues. Inorganic Hg, the predominant species present in maternal milk (Sundberg et al. 1999), is poorly absorbed (< 10%) from the gastrointestinal tract (Elinder et al. 1988).

The accumulation of dietary Hg was inversely proportional to the tissue Hg content at weaning. The highest Hg accumulation was in weanlings with the lowest tissue Hg levels (i.e., controls followed by the 1 and 2 mg/m^3^ concentration groups). Hg accumulation in the 4 mg/m^3^ group did not increase significantly after weaning and was maintained at a relatively constant level. By PND90, the tissue Hg levels in neonates from the control and 1 and 2 mg/m^3^ groups had increased and appeared to be equilibrating at levels present in the 4 mg/m^3^ group. Significantly, this steady-state level was similar to Hg concentrations we measured in tissues of unexposed adult rats fed the same diet (Morgan et al. 2002).

It is unclear whether the Hg concentrations retained in fetuses and neonates had an adverse effect on neural development. Hippocampal, cerebellar, and cortical structures and neuronal interconnections are being formed during the time between birth and weaning (Bayer et al. 1993), the same period when the Hg content of neonatal brain was found to be elevated relative to controls from unexposed dams. Previously, we evaluated the potential effects of gestational Hg^0^ vapor exposure (4 mg/m^3^) on neural function in neonates using a battery of electro-physiologic measures (Herr et al. 2004). In spite of a significant accumulation of Hg in the fetal brain, gestational exposure to Hg^0^ vapor did not cause measurable changes in responses evoked from peripheral nerves or the somato-sensory, auditory, or visual modalities of 20- to 22-week-old neonates. It is possible that these end points were not sufficiently sensitive to detect subtle adverse effects of Hg on the nervous system. Gestational exposure to Hg may have induced protective systems (e.g., metallo-thionein) that allowed neonates to adapt better to the increased Hg levels. It is also possible that by 20–22 weeks after exposure, the animals were able to compensate for any developmental deficits caused by *in utero* Hg exposure.

These studies demonstrated that exposure of pregnant rats to high concentrations of Hg^0^ vapor resulted in a significant accumulation of Hg in brain, liver, and kidney of newborn rats. There was no apparent elimination of Hg from neonatal tissues between birth and weaning; thus, neonates were exposed to elevated levels of Hg during an important period of growth and development. However, a much greater increase in neonatal Hg accumulation occurred after weaning and was attributed to consumption of a diet containing trace levels of methylmercury. By PND90, the Hg body burden had equilibrated at levels found in unexposed adult rats fed the same diet. These data indicate that dietary exposure to trace amounts of methylmercury can result in a significantly greater accumulation of Hg in neonates than gestational exposure to high concentrations of Hg^0^ vapor.

## Figures and Tables

**Table 1:** Concentration of Hg (ng/g) in milk from dams exposed to Hg0 vapor on GD6–GD15.

Hg^0^ exposure (mg/m^3^)	PND4	PND10	PND14	PND21
1	4.16 ± 0.79 (5)	0.93 ± 0.08 (4)	< MDL (5)	1.21 ± 0.07 (5)
2	10.74 ± 1.28 (5)	3.26 ± 0.30 (5)	3.08 ± 0.40 (5)	3.35 ± 1.79 (5)
4	22.59 ± 1.58 (3)	9.73 ± 1.09 (4)	6.33 ± 0.67 (4)	5.05 ± 1.02 (4)

Values shown are mean ± SE (number of dams). The MDL for Hg in milk was 0.663 ng/g. No detectable Hg was found in milk from control dams at any time point.

**Table 2:** Amount of dietary Hg accumulated by neonatal rats fed the NIH-07 diet between weaning and PND90.

Hg^0^ exposure (mg/m^3^)	Brain	Liver	Kidney
0	10	22	32
1	3	8	12
2	1^a^	4	9
4	1^a^	2	3

Values represent the fold increase in tissue Hg content (ng Hg/organ).

a No change.
